# Shorter lifetime of a soil invertebrate species when exposed to copper oxide nanoparticles in a full lifespan exposure test

**DOI:** 10.1038/s41598-017-01507-8

**Published:** 2017-05-02

**Authors:** Micael F. M. Gonçalves, Susana I. L. Gomes, Janeck J. Scott-Fordsmand, Mónica J. B. Amorim

**Affiliations:** 1 Department of Biology & CESAM, University of Aveiro, 3810-193 Aveiro, Portugal; 2 Department of Bioscience, Aarhus University, Vejlsovej 25, PO Box 314, DK-8600 Silkeborg, Denmark

## Abstract

Toxicity tests that last the all life duration of the organisms are not common, instead, long-term tests usually include one reproductive cycle. In the present study we optimized and propose a lifespan (all life) term test using *Enchytraeus crypticus* (Oligochaeta). The effect of copper oxide nanoparticles (CuO-NPs) was assessed in this lifespan test and compared to copper salt (CuCl_2_), using the same effect concentrations on reproduction (EC_50_). Monitored endpoints included survival and reproduction over-time (202 days). Results from survival showed that CuO-NPs caused shorter life of the adults compared to CuCl_2_ (control LT_50_: 218 days > CuCl_2_ LT_50_: 175 days > CuO-NPs LT_50_: 145 days). The effect was even more amplified in terms of reproduction (control ET_50_: 158 days > CuCl_2_ ET_50_: 138 days > CuO-NPs ET_50_: 92 days). Results suggest that CuO-NPs may cause a higher Cu effect via a *trojan horse* mechanism. The use of lifespan tests brings a novel concept in soil ecotoxicity, the longevity. This is a particularly important aspect when the subject is nanomaterials toxicity, where longer term exposure time is expected to reveal unpredicted effects via the current short/long-term tests. The present study confirms this higher effect for CuO-NPs.

## Introduction

Organisms’ longevity is a complex process that can be influenced by various environmental events^1^. Long-term studies such as lifespan tests are very important because many effects cannot be predicted based on short term tests, at least not yet given the shortage level of information^2^. Chemicals’ toxicity is commonly assessed during a certain period of organisms’ lifespan, see e.g. OECD guidelines^3^. In terms of risk assessment, lifespan tests represent a continuous exposure to toxicants during the whole life, which is similar to what can occur in the natural environment, thus providing a highly relevant scenario^4, 5^. There are very few studies with a lifespan range and the species used include *Mus musculus*, *Drosophila melanogaster*, *Saccharomyces cerevisiae* and *Caenorhabditis elegans* ^6^, but does not include any soil dwelling invertebrate. Most of those studies were performed to discover genetic, environmental and pharmacologic modulators of aging for the lifespan extension purpose, providing new insights for human therapy^7, 8^. Studies that assess the effects of contaminants in lifespan are still limited, the few examples use *C. elegans* to investigate lifespan effects of metals, detergents^9, 10^ and nanomaterials (NMs), e.g. silica-nanoparticles (−NPs)^11^. Longer term studies have been showing that time of exposure can highly influence the extent of effects, e.g. in *Mytilus californianus* mussels, exposure to fluoxetine for 47 and 107 days showed effects not observed in the typical 30 days test: biomass decrease^12^. In addition, age is another factor not included in standard testing, for instance as reported for *Enchytraeus crypticus* older worms (3 months old) were more sensitive and have less capacity to recover to okadaic acid exposure than younger worms (25 days old)^13^.

Effects of NMs have been investigated for ca. 2 decades, and there has been increasing alert regarding the need for longer term exposure tests due to the potential long term stability and effects of NMs. So far, results on acute toxicity on e.g. aquatic organisms produced the classification, of Ag-NPs as ‘extremely toxic’ and CuO-NPs as ‘very toxic’^14^. However, most of the data generated so far is on short-term/acute effects, which does not necessarily inform on longer term effects. For instance, Diez-Ortiz *et al*.^15^ found that 52 weeks aged Ag-NPs in LUFA 2.2 soil were more toxic to *Eisenia fetida* than Ag-NPs freshly spiked soil (1 week aged) (reproduction EC_50_ of 34 and 1420 mg Ag/kg, respectively). Also, Waalewijn-Kool *et al*.^16^ reported that the release of Zn ions to soil, from ZnO-NPs, continued over one year, which caused a decrease in toxicity to *Folsomia candida* at that time. In fact, the need of more long-term toxicity studies to obtain a better understanding of NMs effects is fully recognized and pointed out as a current gap and future priority in the knowledge on nanotoxicology^2, 17, 18^.

Copper oxide nanoparticles (CuO-NPs) are used in a wide range of industrial and commercial applications^19–23^. The increased production of CuO-NPs escalates the likelihood of their introduction into the environment. Currently, most of the information regarding the ecotoxicity of CuO-NPs is based on “short-term”/acute effects, mostly in the aquatic compartment (e.g. refs 24–29) and fewer in the soil compartment, e.g. on plants^30–32^ and on soil dwelling invertebrates^33–38^. Therefore, NMs potential long-term toxicity, combined with a likely long residing time, should not be ignored and it is important to investigate the toxicity of these materials^39, 40^.

In the present study we propose a lifespan test i.e., all life duration for the soil living oligochaete *E. crypticus*, until it dies of age. *E. crypticus* is a model standard species where many endpoints can be assessed: survival and reproduction^3, 41^, bioaccumulation^42^, embryo development^43^, or via a full life cycle with hatching, growth, maturity^44^ (see further explanation in the discussion).

The procedures for a lifespan test using *E. crypticus* were optimized using control conditions (un-spiked soil) by monitoring the survival and reproduction of the organism over the entire time of its lifespan. Further, the developed assay was used to study the longevity effects of CuO-NPs in comparison to CuCl_2_, using similar 50% effect concentrations on reproduction (EC_50_ = 1400 and 180 mg Cu/kg for CuO-NPs and CuCl_2_, respectively)^45^.

## Results

### Lifespan assay: optimization in control conditions

**Figure 1:**
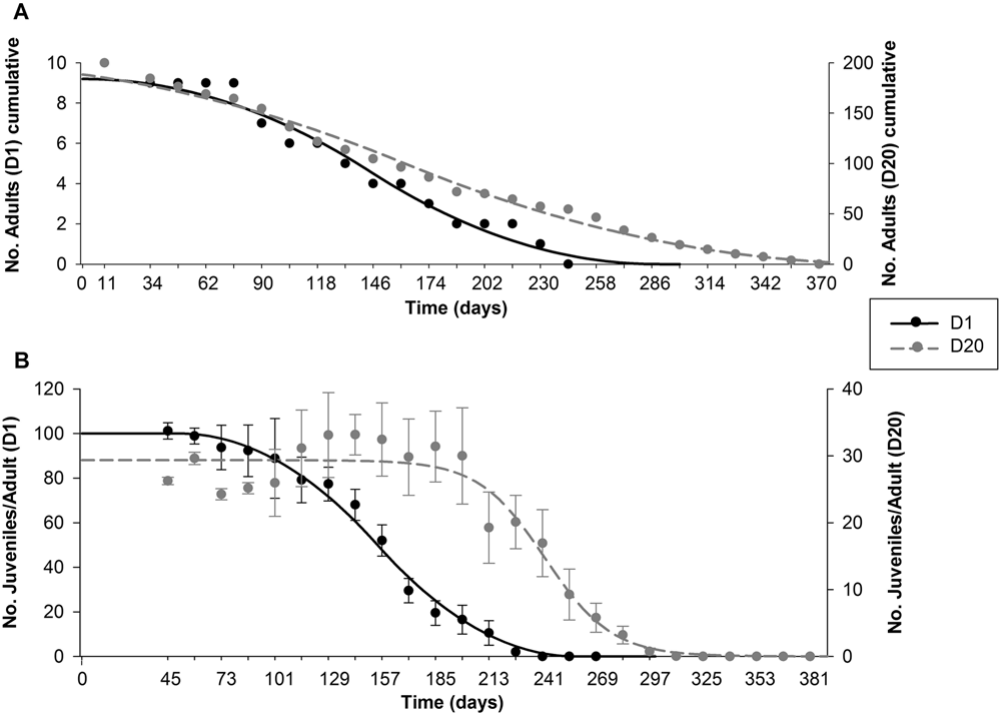
Lifespan test of *Enchytraeus crypticus* at two different organisms’ densities (1 organism (D1) and 20 organisms (D20)) in LUFA 2.2 soil, over-time. (**A**) Adults survival; all values are expressed as cumulative number (N = 10). (**B**) Reproductive output; all values are expressed as average ± standard error (N = 10). The lines represent the model fit to data.

Results on survival at D1 and D20 are shown on Fig. 1A and the ETx values are summarized in Table 1.

**Table 1:** Summary of the Effect Time (ETx) for survival (LTx) and reproduction for *Enchytraeus crypticus* in control conditions in LUFA 2.2 soil at two different organisms’ densities (1 organism (D1) and 20 organisms (D20)).

	Survival				
	LT_10_	LT_20_	LT_50_	LT_80_	Model & parameters
D1	62	92	145	183	Threshold 2P
	(49 < CI < 81)	(80 < CI < 103)	(137 < CI < 152)	(171 < CI < 194)	(S:0.007; Y0:9.2)
D20	26	72	162	227	Threshold 2P
	(14 < CI < 39)	(63 < CI < 80)	(157 < CI < 167)	(219 < CI < 235)	(S:0.004; Y0:200)
	**Reproduction**				
	**ET**_**10**_	**ET**_**20**_	**ET**_**50**_	**ET**_**80**_	**Model & parameters**
D1	97	117	155	182	Threshold 2P
	(78 < CI < 117)	(102 < CI < 131)	(142 < CI < 168)	(160 < CI < 204)	(S:0.010; Y0:100)
D20	204	218	242	267	Logistic 2P
	(180 < CI < 229)	(201 < CI < 236)	(23 < CI < 254)	(249 < CI < 285)	(S:0.014; Y0: 29.3)

95% Confidence Intervals (CI) are shown in brackets.

The lifespan at D1 is lower than at D20 (D1 LT_50_: 145 days, D20 LT_50_: 162 days). Results in terms of reproduction can be observed in Fig. 1B: the number of juveniles produced per adult at D1 is higher than at D20, during the first 101 days, but D1 has a reproduction EC_50_ earlier than D20 (e.g. D1 ET_50_: 154 days, D20 ET_50_: 242 days, Table 1).

### Lifespan assay: exposure to CuO-NPs and CuCl_2_

**Figure 2:**
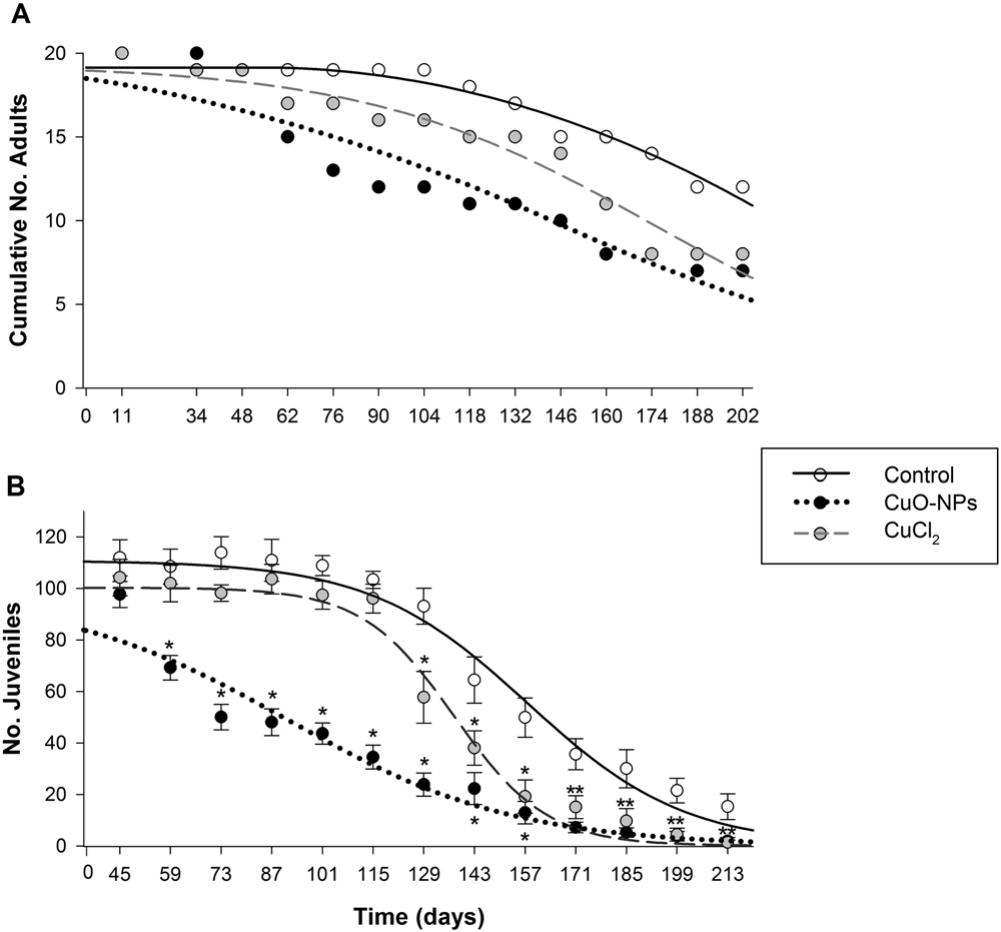
Lifespan test of *Enchytraeus crypticus* when exposed to EC_50_ CuO-NPs and CuCl_2_ (mg Cu/kg DW soil) in LUFA 2.2 soil, over-time. (**A**) Adults survival; all values are expressed as cumulative number (N = 20), (**B**) Reproductive output; all values are expressed as average ± standard error (N = 20). Asterisks indicate significant differences between control and treatments at each sampling day (p < 0.05 Tukey Test or Dunn’s method). The lines represent the model fit to data.

The effects of CuO-NPs and CuCl_2_ on *E. crypticus* lifespan (survival) can be depicted in Fig. 2A and the ETx calculated are summarized in Table 2.

**Table 2:** Summary of the Effect Time (ETx) for survival (LTx) and reproduction for *Enchytraeus crypticus* when exposed to Cu (1400 and 180 mg Cu/kg DW soil for CuO-NPs and CuCl_2_, respectively) in LUFA 2.2 soil.

	Survival				
	LT_10_	LT_20_	LT_50_	LT_80_	Model & parameters
Control	127	157	218	260	Threshold 2P
	(117 < CI < 173)	(151 < CI < 164)	(207 < CI < 227)	(242 < CI < 278)	(S:0.006; Y0: 19.1)
CuCl_2_	77	113	175	237	Logistic 2P
	(59 < CI < 95)	(102 < CI < 125)	(167 < CI < 184)	(218 < CI < 256)	(S:0.006; Y0:19.3)
CuO-NPs	23	64	145	204	Threshold 2P
	(n.d.)	(40 < CI < 88)	(130 < CI < 160)	(175 < CI < 233)	(S:0.005; Y0:19.7)
	**Reproduction**				
	**ET**_**10**_	**ET**_**20**_	**ET**_**50**_	**ET**_**80**_	**Model & parameters**
Control	110	128	158	188	Logistic 2P
	(99 < CI < 121)	(120 < CI < 135)	(153 < CI < 163)	(179 < CI < 197)	(S:0.012; Y0:110.9)
CuCl_2_	110	120	138	155	Logistic 2P
	(101 < CI < 118)	(114 < CI < 126)	(134 < CI < 142)	(149 < CI < 162)	(S:0.020; Y0:100.23)
CuO-NPs	23	48	92	135	Logistic 2P
	(10 < CI < 36)	(39 < CI < 57)	(86 < CI < 97)	(125 < CI < 145)	(S:0.008; Y0:97.7)

n.d. = not determined. 95% Confidence Intervals (CI) are shown in brackets.

CuO-NPs exposure caused a more severe lifespan decrease than CuCl_2_: control LT_50_: 218 days > CuCl_2_ LT_50_: 175 days > CuO-NPs LT_50_: 145 days. Results in terms of reproduction (Fig. 2B) show that CuO-NPs exposure caused higher effects on reproduction in *E. crypticus*, with a 50% reduction in reproduction occurring earlier than for CuCl_2_ (e.g. control ET_50_: 158 days > CuCl_2_ ET_50_: 138 days > CuO-NPs ET_50_: 92 days, Table 2). Comparing the survival and reproduction curves (Fig. 2) it seems that the variation around the model for survival is reflected in the variation around the model for reproduction.

### *In situ* characterisation

The total Cu measured in the soil was ca. 100% of the added total concentration for both CuO-NPs and CuCl_2_. The total Cu in soil solution was less than 1% of the total for CuO-NPs and less than 3% for CuCl_2_. The free active Cu was less than 0.001% for both Cu forms exposure. For controls, the total Cu in soil solution was 0.07% and the active Cu was 0.004%.

## Discussion

This is the first study where the entire lifespan of an enchytraeid was monitored in soil. Previous knowledge on enchytraeids’ lifespan (in agar media) showed: 120 days for *Enchytraeus albidus* ^46^, 127 days for *Enchytraeus doerjesi* ^47^ and 224 days for *Enchytraeus coronatus* ^48^. Westheide and Graefe^47^ also reported an 85 days lifespan for *E. crypticus* which is considerably less than the 244 and 370 days we observed for D1 and D20, respectively. Possibly, the differences in terms of test media, soil (in our study) and agar media^47^ influence the longevity, as the food source was the same (oats). Hence, this indicates that *E. crypticus* can live longer in soil compared to agar.

The experimental test design as proposed here can be used as draft for a lifespan test in soil for *E. crypticus*. Results showed that the selected sampling points to assess the survival and reproductive output over-time were adequate. Although it is a very long duration of the test, the associated material costs are relatively low, except in terms of time consumption and human resource, but the level of information is very high. The majority of the studies that assess endpoints like survival, reproduction, bioaccumulation or growth are based on shorter exposure periods, covering up to 6 weeks of duration^3, 42^ and cannot predict the effects on longevity.

Analysis of organisms’ survival over time showed that at D1 enchytraeids died earlier compared to D20. The reproductive output (number of juveniles per adult) was higher for the D1 than for the D20, which is in agreement with results from a detailed density study using the same species^49^. This has also been observed in other studies: lower reproductive output at higher densities compared to lower densities, for instance in *Lumbricus terrestris* the cocoon production was 1.5, 0.6, 0.1, 0.06, 0.04 and 0.0 at D1, D2, D3, D4, D6 and D8, respectively^50^.

Regarding reproduction, the observed decrease in reproductive output over time is possibly age related. Changes in fertility in relation to age (reproductive senescence) have been reported in many organisms^51^. For example, in *Caenorhabditis elegans* the fast decline in the reproduction begins at young to middle age due to sperm depletion^52^ whereas in *Drosophila melanogaster* is due to apoptosis of ageing egg chambers^53^. In *E. crypticus* the reproductive output showed a variation along the lifespan of the organisms and decreased with aging. This further reiterates the importance of using organisms with synchronized age in ecotoxicological testing as recommended for this species and implemented in the full life cycle test^44^.

Results showed that for the same reproduction EC_50_ CuO-NPs were more toxic than CuCl_2_, i.e. exposure to CuO-NPs caused shorter longevity and lower reproduction. Please note that results reflect a comparison between reproduction EC50s (not the same or a range of soil concentrations) which will provide the most stable measure for the distance between the concentration curves. To test more concentrations would be very interesting to study as well, and would illustrate whether the steepness of the concentration-response curves are the same. However, the importance in the present study was the distance between the curves, hence to optimise time and resources we have selected this reduced design. Regarding the longevity (survival), maybe the mechanism is similar to what is reported in *Mytilus galloprovincialis* ^54, 55^, i.e. CuCl_2_ was easily eliminated whereas CuO-NPs had slower elimination rate resulting in an increased accumulation with time of exposure. In short, even though Cu concentrations in the digestive gland of mussels were higher for CuCl_2_ than for CuO-NPs in the first week of exposure, Cu accumulation decreased for CuCl_2_ at the end of experiment (15 days) whereas it increased for CuO-NPs exposure. In the present work, the soil was spiked every 15 days, which means a continuous renewal or new pulse of Cu source. The following may be considered: (a) only dissolution and transformation of the NPs over 15 days is monitored and can have an effect in the test and (b) if NPs within the organism are refilled by this approach and we have NP-specific effects only in the early time of exposure because the NPs degrade quickly, then the refilling could be amplifying the NP effect compared to one long term exposure without renewal. Hence, the observed differences in terms of longevity (59–143 days period) could possibly be related with different accumulation/elimination rates between NPs and salt and less likely due to the decrease of Cu bioavailability by sorption to soil over time.

From day 157 onwards, effect levels became similar between CuO-NPs and CuCl_2_, which could mean that, after prolonged exposure, Cu elimination (from CuCl_2_) was less efficient (also linked to the age of the organisms, note that from day 143 there is a reduction in reproduction, also in control) and the effects caused by CuCl_2_ meet those caused by CuO-NPs. Alternatively or additionally, the reason could also be the general deteriorating health of the adults.

The mechanism of Cu uptake from CuO-NPs is not fully understood. Some authors explain the higher cytotoxicity of CuO-NPs (in comparison to CuCl_2_ on a mass basis) via a *trojan horse* mechanism, i.e. NPs can release a boom of metal ions inside the cells, possibly due to lower pH which causes a higher dissolution^56, 57^. Shi *et al*.^30^ reported higher toxicity of CuO-NPs (in comparison to CuCl_2_) to *Landoltia punctate* due to the high uptake of ions released from the NPs, but question the intra-cellular form of Cu and if the CuO-NPs themselves are taken up into the cells. Pradhan *et al*.^27^ suggest the intake of CuO-NPs in *Allogamus ligonifer*, and also state that the Cu ions released from the CuO-NPs may contribute to the toxicity of CuO-NPs. A study by Navratilova *et al*.^58^ showed that it was possible to detect larger CuO-NPs by Single Particle ICP-MS, but due to the interaction with soil components it was very difficult (or impossible) to separate Cu^+^ bound to small natural particles from CuO-NPs present in the sample. The CuO-NPs used in the present study were below the theoretical detection limit, so it was not possible to detect them. However, in their study Navratilova *et al*.^58^ indicated that CuO-NPs persisted in the nano form (even though in the form of agglomerates) and do not completely solubilize in the presence of soil components, i.e. organic matter. Hence, in the current work, the exposure was based on measurement of total and active Cu in the soil and soil solution. Nevertheless, it was not possible to directly explain the difference in toxicity based on the measured concentrations in soil solution, i.e. total and active ions. Although, it was observed that the ratio between the total Cu concentration in soil solution of the CuO-NPs and of the CuCl_2_ was similar to the ratio between the LT_50_s for reproductive output of the two Cu-forms. For survival the same was true for the measured active ions i.e. a similar ratio. This however may be more a coincidence than a confirmation of a causative phenomenon, but deserve further consideration when broadly available and validated techniques for discrimination between ionic and particle based forms in solid media exist^59^. Additionally, a full dose response design to compare CuCl_2_ and CuO-NPs in a life span test would have advantages and possibly reveal further on the differences in toxicity mechanisms i.e. if the dose-response curves shows different steepness. Using a full dose-response design would enable us to follow the ECx at each time point, and see whether this has intermediate changes. The same refers to the chemicals life cycle, where a testing with materials aging along the test duration (compared to continuous pulse) would allow broader interpretation and discussion, adding relevancy.

## Conclusions

A lifespan test was developed for the first time in soil organisms and includes longevity as an additional endpoint for ecotoxicology. The proposed lifespan term test will be extremely useful to assess the prolonged effects of toxicants, e.g. very relevant for nanomaterials. Results showed precisely that longevity was more affected for CuO-NPs compared to CuCl_2_ exposure (when tested at a similar reproduction effect concentration, EC_50_), which would not be predictable based on the current standard test. We understand that the test length may be an issue but highly recommend the performance of longevity test for selected cases and design, in particular for the testing of nanomaterials.

## Materials and Methods

### Test organisms

The test organism belongs to the species *Enchytraeus crypticus*, Westheide and Graefe, 1992. Cultures were kept in agar plates fed ad libitum with grinded and autoclaved oats and maintained in laboratory under controlled conditions, e.g. photoperiod of 16:8 hours (light: dark) and temperature of 20 ± 1 °C. Juveniles of synchronized age (11 days) were used. For details on culture synchronization see Bicho *et al*.^44^.

### Test soil

The standard natural soil LUFA 2.2 (Speyer, Germany) was used. Main properties of the soil can be summarised as follows: pH (0.1 M CaCl_2_) of 5.5, 43.3% of maximum water-holding capacity (WHCmax), 1.61% organic carbon and a particle size distribution of 7.9% clay, 16.3% silt and 75.8% sand.

### Test procedures

#### Development of the lifespan assay: control conditions

The development/optimization of the lifespan assay was done in un-spiked soil, moistened to 50% of the WHCmax. Juveniles of synchronized age (11 days) were randomly selected and placed in each well (of the 6-well plates) at two densities: 1 (D1) and 20 (D20) organisms per replicate, ten replicates were used. After 25 days (11 plus 14 days to allow growth and reaching maturity) adults’ survival was recorded and the surviving adults (25 days old) were transferred to new test plates, in the same conditions, i.e. D1 or D20, respectively. To ensure that juveniles were not transferred together with the adults, prior the transference to the new test plates, the organisms were cleaned in a petri-dish with distilled water and checked under a stereo microscope (Zeiss Stemi 2000-C). Every 15 days, the survival of the adults was recorded and the surviving adults were transferred to new test plates as described above. After each transfer, the previous test plates were left during 11 more days to ensure that the cocoons laid have time to hatch; after that, the soil in the well plates was transferred to glass vials and fixated with 96% ethanol and Bengal rose (1% in ethanol) and the juveniles were counted using a stereo microscope (Zeiss Stemi 2000-C).

Food (grinded and autoclaved oats) was added weekly (2 and 10 mg for D1 and D20 exposed organisms, respectively). Water was added every 3 days. The test was maintained at a photoperiod of 16:8 hours light:dark and at 20 ± 1 °C. The test ran until all the adults were dead (370 days).

#### Lifespan assay: exposure to CuO-NPs and CuCl_2_

For the test with CuO-NPs and CuCl_2_, organisms (juveniles of synchronized age) were exposed at density D1 following the procedures described above. Density D1 was chosen due to the increased power of traceability of results to one individual (compared to an average, etc.); besides, there is lower variability in terms of reproductive output compared to D20. Twenty (20) replicates per test condition were used, 2 mg of food was added weekly and water adjusted every 3 days. The test was maintained at a photoperiod of 16:8 hours light:dark and at 20 ± 1 °C. The test ran for 202 days (plus 11 more days to allow the cocoons to hatch); the test duration was selected based on the results from the optimization of the lifespan assay in control conditions (≈LT_80_).

**Figure 3:**
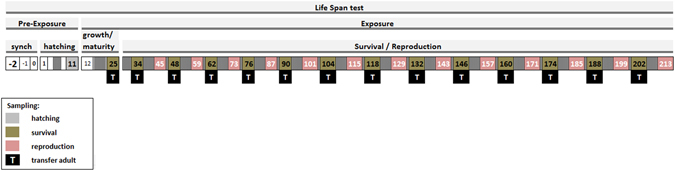
Schematic representation of the proposed Lifespan test for *Enchytraeus crypticus*, including the pre-exposure period (synch and hatching), the sampling days and endpoints evaluated (survival and reproduction) over the test duration.

The schedule, test design and sampling days, as optimized, are presented in Fig. 3.

#### Test chemicals and spiking

Copper-salt (CuCl_2_•2H_2_O) and Copper Oxide Nanoparticles (FP7 SUN pristine materials) were used (Table 3). The CuCl_2_ was obtained from Sigma-Aldrich (CAS number 10125-13-0) with a purity of 99%. The tested concentrations were selected based on the EC_50_ for reproduction effect (CuCl_2_ = 180 mg Cu/kg and CuO-NPs = 1400 mg Cu/kg soil dry weight) as known from previous Enchytraeid Reproduction Test (ERT) results^45^. CuCl_2_ was added to pre moistened soil (20% w/w) as aqueous solution. For CuO-NPs, the NPs were added as dry powder to the soil as recommend by OECD for the testing of insoluble substances^60^. In short, CuO-NPs were thoroughly mixed manually with the dry soil to obtain the corresponding concentration. After that, deionized water was added to reach 50% of the soil WHC. All soils were homogeneously mixed and allowed to equilibrate for 1 day before test start. Soil was spiked and renewed every 15 days during sampling.

**Table 3:** Characteristics of the tested CuO-NPs (Source: FP7-SUN project).

Characteristics	CuO-NPs
Manufacturer	Plasma Chem
CAS number	1317-38‐0
Primary size distribution (average)	3–35 (12)
Mode (1st quartile - 3rd quartile)[nm]	10 (9.2–14)
Shape	Semi-spherical
Average crystallite size [nm]	9.3
Crystallite phases (%)	Tenorite 100%
Dispersability in water: D50 [nm];	139.5 ± 4.6;
average agglomeration number (AAN)	346
Dispersability in modified MEM: D50 [nm];	85.2 ± 2.7;
average agglomeration number (AAN)	77
Z‐potential in UP water [mV]	+28.1 ± 0.6
Isoelectric point [pH]	10.3
Photocatalysis: photon efficiency [unitless]	1.5 × 10^−4^
Specific Surface Area [m^2^ g^−1^]	47.0 ± 1.7
Pore sizes [nm]	13.5 ± 1.6 (BJH)
	23.0 ± 0.9 (AVG)
Surface chemistry [atomic fraction]	Cu = 0.46 ± 0.05; O = 0.47 ± 0.05
	C = 0.07 ± 0.01
Chemical impurities [mg kg^−1^]	Na: 505 ± 30; Pb: 36 ± 2 Ag: 13 ± 4

Controls correspond to un-spiked LUFA 2.2 soil moistened until 50% of WHC. Test vessels consisted of 6-well plates (35 mm ø), each well containing 5 g of moistened soil (50% of WHC). Treatments and replicates were distributed randomly in the test plates.

#### *In situ* characterisation

The amount of Cu was measured in the test soil and in soil solution (for method details see Gomes *et al*.^61^) in a concurrent experiment over 28 days. In the soil the total Cu was measured (by Graphite Furnace Atomic Absorption Spectroscopy: AAS-GF). In soil solution, both the total Cu and free active form were measured by AAS-GF and by ion-selective electrode, respectively. The CuO present as nanomaterials was not determined in the soil, due to the technical difficulties e.g. that the particle size is below the theoretical detection limit of 15 nm (see Navratilova *et al*.^58^).

#### Data analysis

To assess significant differences between treatments at each sampling day One-Way ANOVA (using Tukey Test or Dunn’s method for multiple comparisons) was used (SigmaPlot 11.0).

Lethal Time (LTx) as time to reduce survival in x% and Effect Time (ETx) as time to reduce reproduction in x% calculations were performed for survival and reproduction, respectively, using the logistic equation or threshold sigmoid 2 or 3 parameters regression models (TRAP 1.30 software).
